# Induction of differentiation of intrahepatic cholangiocarcinoma cells to functional hepatocytes using an organoid culture system

**DOI:** 10.1038/s41598-018-21121-6

**Published:** 2018-02-12

**Authors:** Yoshimasa Saito, Toshiaki Nakaoka, Toshihide Muramatsu, Hidenori Ojima, Aoi Sukeda, Yuko Sugiyama, Ryoei Uchida, Ryo Furukawa, Aya Kitahara, Toshiro Sato, Yae Kanai, Hidetsugu Saito

**Affiliations:** 1 Division of Pharmacotherapeutics, Keio University Faculty of Pharmacy, 1-5-30 Shibakoen, Minato-ku, Tokyo, 105-8512 Japan; 2 Division of Gastroenterology, Department of Internal Medicine, Keio University School of Medicine, 35 Shinanomachi, Shinjuku-ku, Tokyo, 160-8582 Japan; 3 Department of Pathology, Keio University School of Medicine, 35 Shinanomachi, Shinjuku-ku, Tokyo, 160-8582 Japan; 4 Department of Pathology and Clinical Laboratories, National Cancer Center Hospital, 5-1-1 Tsukiji, Chuo-ku, Tokyo, 104-0045 Japan

## Abstract

Intrahepatic cholangiocarcinoma (IHCC) is a highly aggressive malignancy with a poor prognosis. It is thought to originate from cholangiocytes, which are the component cells of intrahepatic bile ducts. However, as patients with viral hepatitis often develop IHCC, it has been suggested that transformed hepatocytes may play a role in IHCC development. To investigate whether IHCC cells can be converted to functional hepatocytes, we established organoids derived from human IHCC and cultured them under conditions suitable for hepatocyte differentiation. IHCC organoids after hepatocyte differentiation acquired functions of mature hepatocytes such as albumin secretion, bile acid production and increased CYP3A4 activity. Studies using a mouse model of IHCC indicate that Wnt3a derived from macrophages recruited upon inflammation in the liver may promote the malignant transformation of hepatocytes to IHCC cells. The results of the present study support the recently proposed hypothesis that IHCC cells are derived from hepatocytes.

## Introduction

Intrahepatic cholangiocarcinoma (IHCC) is the second most prevalent malignancy affecting the liver^1,2^. Patients with inoperable IHCC generally receive a chemotherapy regimen of gemcitabine and cisplatin. However, the effect of these drugs is limited, and the 5-year survival rates of patients are very low^3–6^. In addition, the lack of *in vitro* models that can reproduce the properties of human IHCC has hindered understanding of its molecular pathogenesis and development of more effective therapeutic drugs.

IHCC is thought to originate from cholangiocytes, which are the component cells of intrahepatic bile ducts. However, patients with chronic hepatitis due to infection with hepatitis B virus and hepatitis C virus sometimes develop IHCC, suggesting that transformed hepatocytes may also give rise to IHCC^7–10^. Indeed, recent studies using a mouse model of IHCC have revealed that hepatocytes were converted to biliary lineage cells during the initiation of IHCC by activation of the Notch signaling pathway^11,12^. Nishikawa *et al*. have also shown that mature hepatocytes can undergo transdifferentiation to bile duct cells within a collagen gel matrix^13,14^. These findings suggest that transdifferentiation of mature hepatocytes to bile duct cells is a critical event in the initiation of IHCC.

LGR5, a member of the Wnt signaling pathway, has been identified as a new molecular marker of stem cells in the small intestine, colon, stomach, liver and pancreas^15–20^. The newly developed technique of three-dimensional (3D) culture known as organoid culture allows LGR5-positive stem cells to form budding cyst-like structures (organoids). This type of 3D culture uses serum-free medium that includes only predefined factors such as R-spondin 1 and epidermal growth factor (EGF). R-spondin 1 has been identified as a ligand for Lgr5 and is an essential factor for activation of the Wnt signaling pathway^21,22^. Thus, interaction between Lgr5 and R-spondin 1 plays a pivotal role in the self-renewal of stem cells. Using this new 3D culture system, organoid models of human colon, prostate and pancreatic cancers have been established^16,23–26^. Organoids derived from tumor tissues closely recapitulate the properties of the primary tumors^25,26^. A recent study has shown that adult bile-duct-derived bipotent progenitor cells can be expanded as epithelial organoids *in vitro* and differentiated into functional hepatocytes after organoid culture in defined medium^27^. To investigate whether IHCC cells can be converted to functional hepatocytes, we established organoids derived from human IHCC and cultured them under conditions suitable for hepatocyte differentiation.

## Results

### Establishment and long-term *in vitro* culture of organoids derived from human IHCC

**Figure 1:**
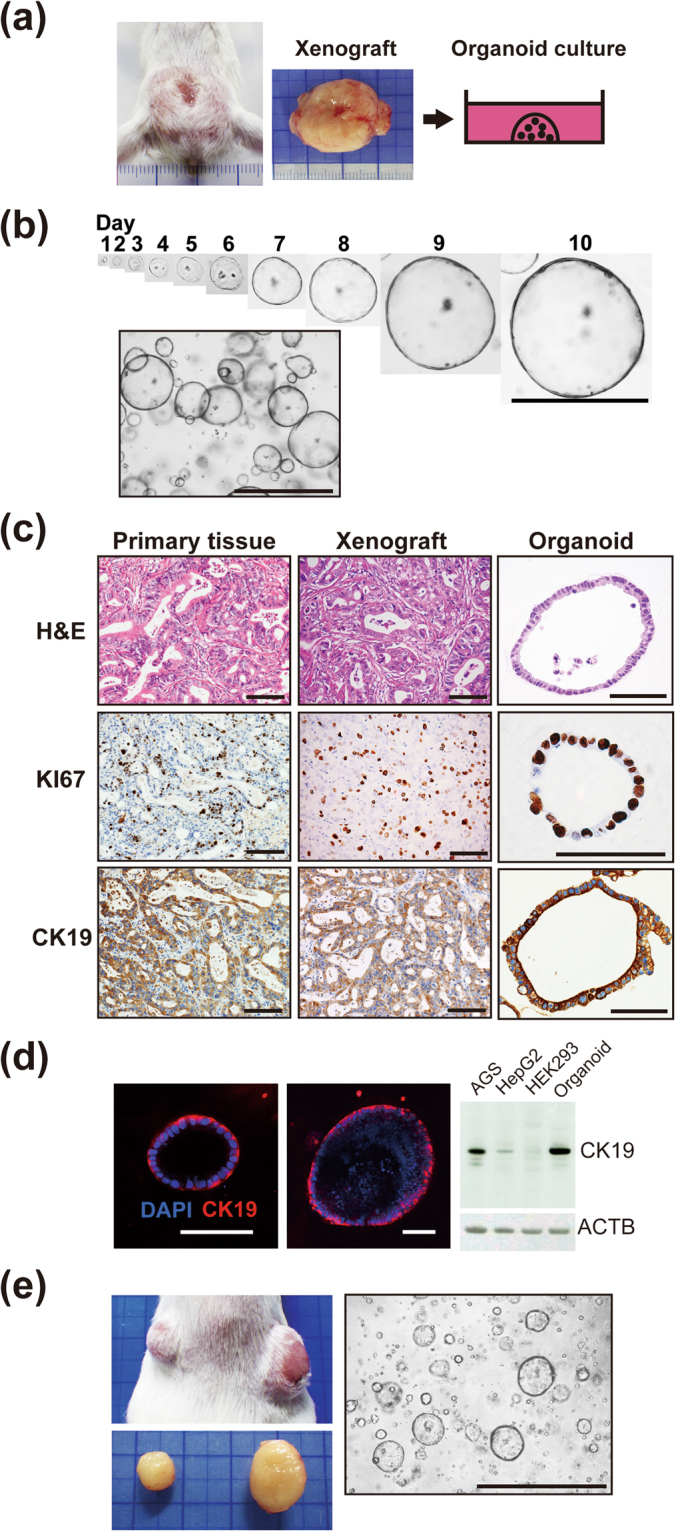
Establishment and long-term *in vitro* culture of organoids derived from human IHCC. (**a**) Macroscopic features of the human IHCC xenograft that had been implanted subcutaneously into a SCID mouse for approximately 3 months. The xenografted tumor was then excised and subsequently subjected to organoid culture. (**b**) Representative serial images of a single cholangiocarcinoma stem cell expanding into cystic organoids, and bright-field images of IHCC organoids. Scale bars: 1000 μm. (**c**) H&E, KI67 and CK19 staining of the primary tissue, xenograft and organoid derived from IHCC. Scale bars: 100 μm. (**d**) Immunofluorescence staining of CK19 (red) in IHCC organoids. DNA is stained with DAPI (blue). Scale bars: 100 μm (left and middle). Western blotting of CK19 in IHCC organoids and the AGS, HepG2 and HEK293 cell lines. β-Actin (ACTB) was used as an internal control (right). (**e**) Subcutaneous implantation of IHCC organoids into a SCID mouse (right: 8 × 10^5^ cells, left: 3 × 10^5^ cells). Two months after implantation, the tumors were excised and further subjected to organoid culture. Scale bar: 1000 μm.

Here we established organoids derived from human IHCC using xenograft tissues and surgically resected specimens from patients with IHCC. The first IHCC organoids were established using cholangiocarcinoma xenograft tissues derived from a 70-year-old female patient with moderately differentiated IHCC^28^. The macroscopic features of the IHCC xenograft that had been implanted subcutaneously into a SCID mouse for approximately 3 months are shown in Fig. 1a. This xenografted tumor was subsequently excised from the mouse and subjected to organoid culture. Representative serial images of single cholangiocarcinoma stem cells expanding into cystic organoids are shown in Fig. 1b. This IHCC organoid gradually expanded and reached a size of approximately 1000 μm by day 10. We have been able to maintain this IHCC organoid stably for over one year (Fig. 1b).

Histopathological examination of the primary IHCC tissue revealed moderately differentiated adenocarcinoma with glandular and tubular structures (Fig. 1c). The xenografted tissue showed histopathological features similar to those of the original primary IHCC, and the IHCC organoid had a monolayered cystic structure, recapitulating the tissue of the original primary IHCC. KI67 is a general marker of cancer cell proliferation, and CK19 is often used as a molecular marker for pathological diagnosis of cholangiocarcinoma. We observed high immunoreactivity for KI67 in the nuclei and for CK19 in the cytoplasm of components cells of the IHCC organoids and tissues (Fig. 1c and d). The primary tissue, xenograft tissue and organoid all showed similar KI67 and CK19 staining patterns (Fig. 1c). The results of Western blotting showed that CK19 was highly expressed in IHCC organoids in comparison with the AGS, HepG2 and HEK293 cell lines, which are derived from gastric cancer, liver cancer and embryonic kidney, respectively (Fig. 1d).

To confirm the tumorigenic capacity of IHCC organoids, we implanted the organoids subcutaneously into the backs of SCID mice (right: 8 × 10^5^ cells, left: 3 × 10^5^ cells). Two months after implantation, the organoids had formed tumors whose size was proportional to the number of cells injected (Fig. 1e). We further removed these implanted tumors and subjected them to organoid culture. As shown in Fig. 1e, cells derived from the implanted tumors formed organoids and expanded under the same culture conditions.

### Induction of differentiation of IHCC cells to functional hepatocytes

**Figure 2:**
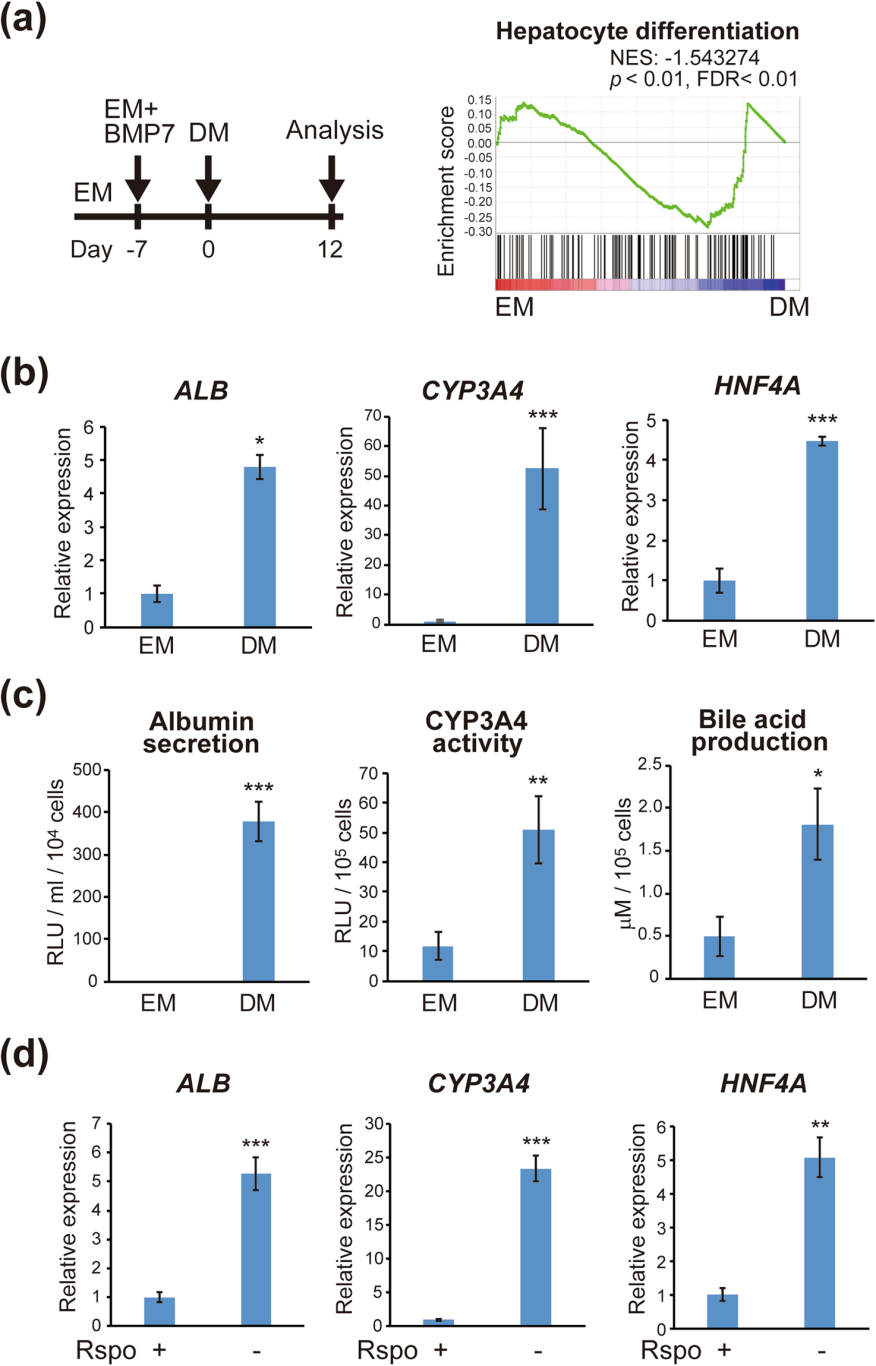
Induction of differentiation of IHCC organoids to functional hepatocytes. (**a**) Schedule for hepatocyte differentiation using IHCC organoids. IHCC organoids were cultured for 7–10 days in EM additionally supplemented with BMP7. Then, the culture medium was changed to DM. DM was changed every 2–3 days for a period of 12 days. GSEA was performed using a ‘hepatocyte differentiation’ gene set. IHCC organoids cultured in DM have a strongly enriched hepatocyte differentiation signature (*p* < 0.01, FDR < 0.01). (**b**) Relative expression of *Albumin* (*ALB*), *CYP3A4* and *HNF4A* in IHCC organoids cultured in EM or DM. (**c**) Albumin secretion, CYP3A4 activity and bile acid production in IHCC organoids cultured in EM or DM. (**d**) Relative expression of *Albumin* (*ALB*), *CYP3A4* and *HNF4A* in IHCC organoids cultured in EM, or in EM without R-spondin 1 (Rspo).

A recent study has shown that liver organoids cultured in differentiation medium (DM) displayed a hepatocyte-like phenotype, whereas those cultured in expansion medium (EM) displayed a bile duct-like phenotype^27^. To investigate whether IHCC cells can be converted to functional hepatocytes, we conducted hepatocyte differentiation using IHCC organoids in a similar manner. A ‘hepatocyte differentiation’ gene set from the expression data was used for GSEA^29^. GSEA of IHCC organoids revealed that IHCC cells cultured in DM showed significant enrichment of the hepatocyte differentiation signature (Fig. 2a, *p* < 0.01, FDR < 0.01).

As shown in Fig. 2b, culture of IHCC organoids with DM induced significant up-regulation of markers of mature hepatocytes such as *Albumin*, *CYP3A4* and *HNF4A*. We performed functional studies of IHCC cells after hepatocyte differentiation by measuring albumin and bile acid secreted into the culture supernatant and CYP3A4 activity. The amounts of albumin and bile acid and CYP3A4 activity were significantly increased in IHCC organoids that had been cultured in DM relative to those that had been cultured in EM (Fig. 2c), indicating that IHCC organoids can reacquire a variety of hepatocyte functions upon culture in DM.

To identify factors essential for acquisition of hepatocyte function, we cultured IHCC organoids in DM after exclusion of one specific factor such as BMP7, FGF19, DAPT or dexamethasone. DAPT is a γ-secretase inhibitor that indirectly inhibits the Notch signaling pathway. As shown in Supplementary Fig. S1a, no specific factor in DM had any significant effect on markers for mature hepatocytes. A recent study has shown that activation of the Wnt signaling pathway suppresses hepatocyte differentiation^27^, suggesting that inhibition of the Wnt signaling pathway is essential for hepatocyte differentiation. As expected, removal of R-spondin 1, the ligand of LGR5 in Wnt signaling, from EM increased the expression of mature hepatocyte markers to a marked degree (Fig. 2d).

**Figure 3:**
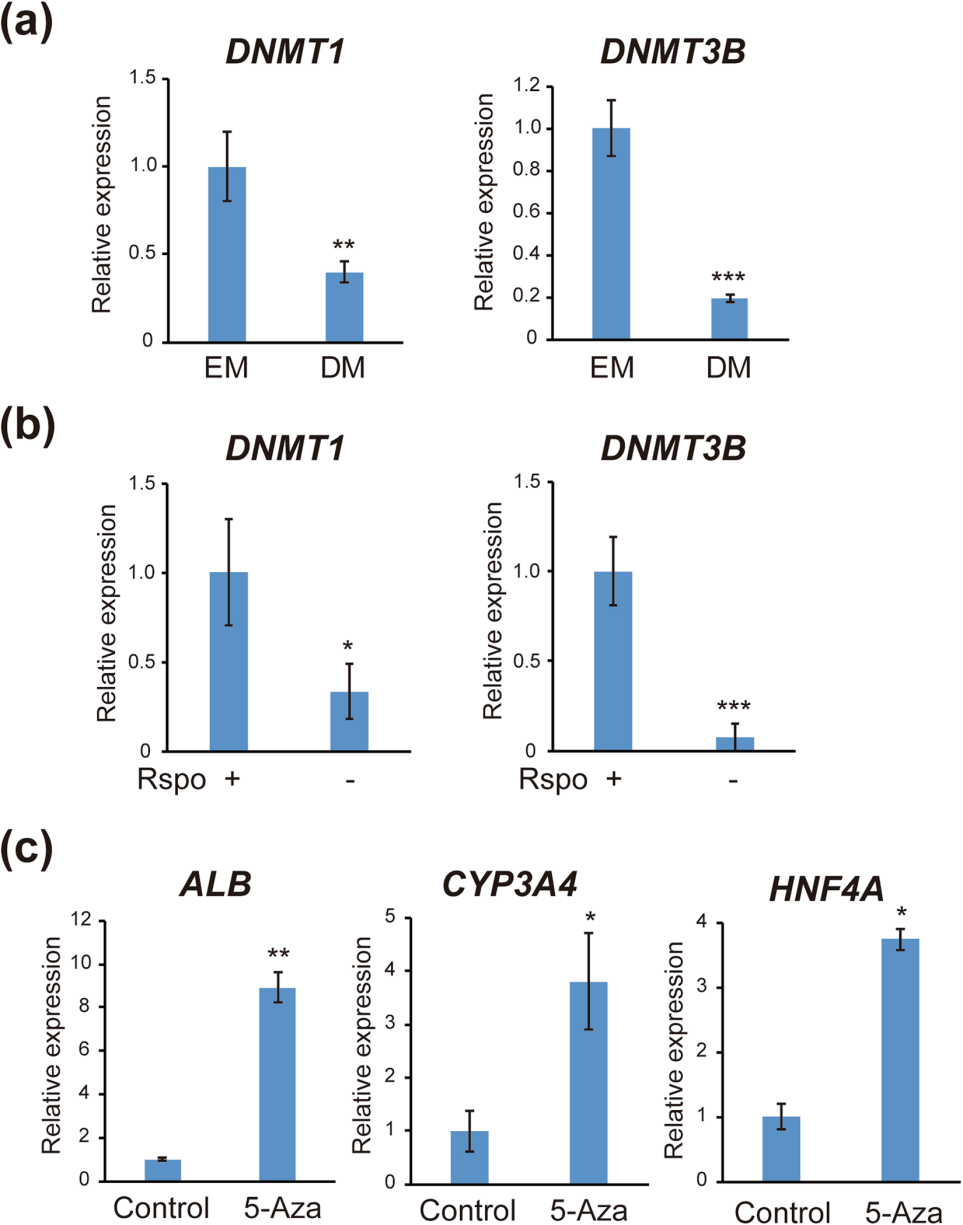
Expression of *DNMT1* and *DNMT3B* during hepatocyte differentiation. (**a**) Relative expression of DNA methyltransferases *DNMT1* and *DNMT3B* in IHCC organoids cultured in EM or DM. (**b**) Relative expression of *DNMT1* and *DNMT3B* in IHCC organoids cultured in EM, or in EM without R-spondin 1 (Rspo). (**c**) Relative expression of *Albumin* (*ALB*), *CYP3A4* and *HNF4A* in IHCC organoids after treatment with 1 μM 5-Aza-CdR (5-Aza).

Epigenetic alterations including DNA methylation play critical roles in cell differentiation. The results of our microarray and quantitative RT-PCR analyses showed that expression of DNA methyltransferases *DNMT1* and *DNMT3B* was markedly suppressed after hepatocyte differentiation (Fig. 3a). Expression of *DNMT1* and *DNMT3B* was significantly decreased after removal of R-spondin 1 from EM (Fig. 3b), indicating that inhibition of Wnt signaling suppressed the expression of DNA methyltransferases. We further treated IHCC organoids with the DNA demethylating agent 5-Aza-CdR. As shown in Fig. 3c, DNA demethylation induced by 5-Aza-CdR significantly increased the expression of markers for mature hepatocytes. These findings suggested that expression of mature hepatocyte markers is under epigenetic control, and that inhibition of DNA methylation may induce differentiation of IHCC cells to mature hepatocytes. We examined the levels of DNA methylation around the promoter regions of genes expressed in mature hepatocytes, including *HNF4A*, by pyrosequencing in IHCC organoids, but found no significant difference in DNA methylation between EM and DM culture (data not shown). We further performed genome-wide analysis of DNA methylation by BeadChip assay. Since the levels of *DNMT1* and *DNMT3B* expression were markedly reduced after hepatocyte differentiation, we screened genes that showed a reduction of DNA methylation in IHCC organoids that had been cultured in DM. The results of the BeadChip assay defined as AVG Beta (EM) > 0.3 and AVG Beta Ratio (EM/DM) > 2.0 are shown in Supplementary Table S2. We found that some genes, including *ALDH3A*, *JAK2* and *miR-505*, showed a decreased level of DNA methylation in IHCC organoids that had been cultured in DM, whereas genes that are associated with hepatocyte differentiation showed no significant difference in DNA methylation.

### Hepatocyte differentiation reduces the malignant potential of IHCC cells *in vitro* and *in vivo*

**Figure 4:**
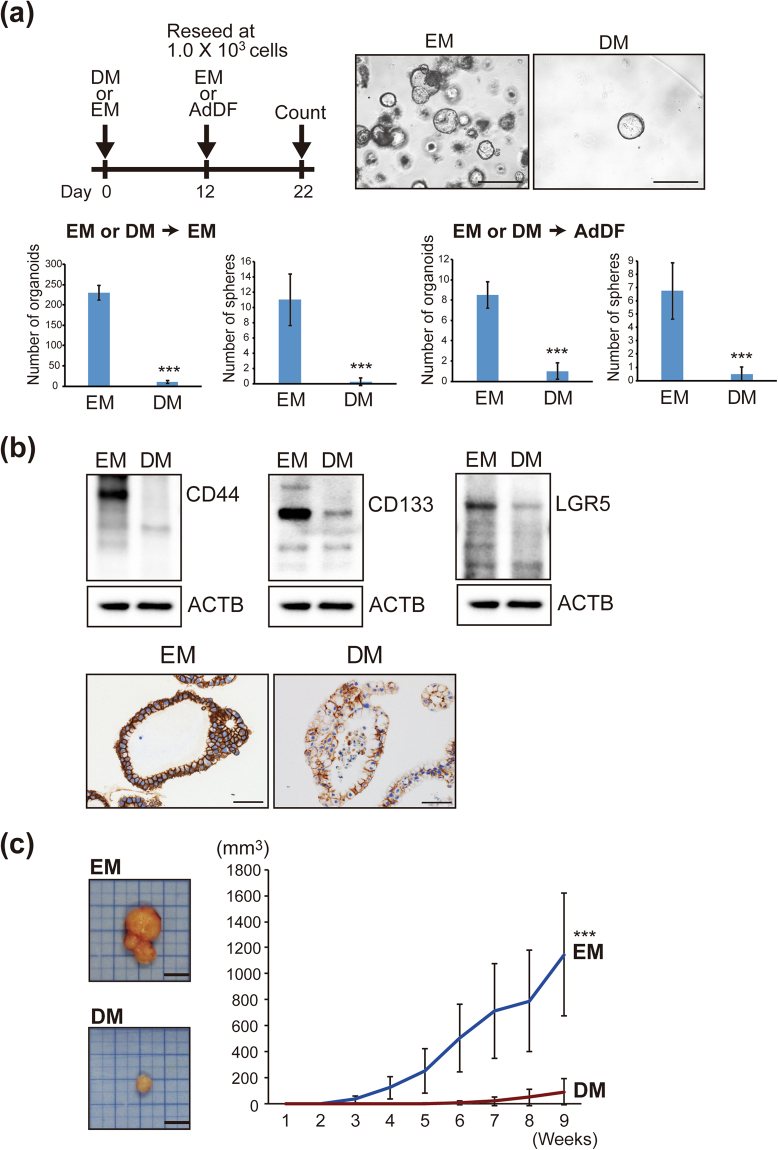
Hepatocyte differentiation reduces the malignant potential of IHCC cells *in vitro* and *in vivo*. (**a**) Schedule for hepatocyte differentiation and counting of organoids and spheres. IHCC organoids were cultured in DM or EM for 12 days, then reseeded at 1.0 × 10^3^ cells and cultured in EM or Advanced DMEM/F12 (AdDF) with 10% FBS for 10 days. The numbers of organoids and spheres were counted. Scale bars: 500 μl. (**b**) Western blotting of the tumor initiating markers CD44, CD133 and LGR5 in IHCC organoids cultured in EM or DM (upper). Immunohistochemical staining of CD44 in IHCC organoids cultured in EM or DM (lower). Scale bars: 50 μm. (**c**) Tumor volumes of xenografted IHCC organoids cultured in EM or DM. We implanted 1 × 10^6^ cells of IHCC organoids cultured in EM or DM subcutaneously into the backs of SCID mice. Tumor volumes of xenografted IHCC organoids cultured in EM (n = 8) or DM (n = 8) on SCID mice were measured.

We next investigated whether hepatocyte differentiation of IHCC cells would reduce the malignant potential of IHCC organoids. The number of organoids would likely be associated with the proliferation of cancer stem cells. To evaluate the malignant potential of organoids with or without hepatocyte differentiation under the same conditions, IHCC organoids cultured in EM or DM were reseeded at 1.0 × 10^3^ cells and cultured in EM or Advanced DMEM/F12 (AdDF) with 10% fetal bovine serum (FBS) for 10 days. The numbers of organoids and spheres were then counted (Fig. 4a). As shown in Fig. 4a, the numbers of spheres and organoids were markedly decreased after culture in DM. In addition, we explored the effect of hepatocyte differentiation on the signatures of cancer stem cells and epithelial-mesenchymal transition (EMT). As shown in Fig. 4b and Supplementary Fig. S2a, the tumor-initiating markers CD44, CD133 and LGR5 were markedly down-regulated after culture of IHCC organoids in DM in comparison to culture in EM. *Snail1*, the transcriptional factor that induces EMT, was also down-regulated after hepatocyte differentiation. The mesenchymal cell marker *Vimentin* was down-regulated and the epithelial cell marker *E-cadherin* was up-regulated by culture in DM, suggesting that hepatocyte differentiation suppresses EMT of IHCC organoids. To identify factors in DM essential for reduction of malignant potential, we cultured IHCC organoids in DM after exclusion of single specific factors such as BMP7, FGF19, DAPT and dexamethasone, and examined the expression levels of *CD44* and EMT markers (Supplementary Fig. S1b). We found that DAPT, an inhibitor of the Notch signaling pathway, was important for suppression of *CD44*, *Sanil1* and *Vimentin*, suggesting that inhibition of Notch signaling is critical for reducing the malignant potential of IHCC organoids.

To confirm these findings *in vivo*, we implanted IHCC organoids that had been cultured in EM or DM (1 × 10^6^ cells) subcutaneously into the backs of SCID mice. We used 16 SCID mice for our xenograft formation assay of IHCC organoids cultured in EM (n = 8) or DM (n = 8). After implantation, the IHCC organoids formed xenograft tumors. As shown Fig. 4c, the volumes of xenografted tumors derived from IHCC organoids that had been cultured in DM were significantly reduced in comparison to those of IHCC organoids that had been cultured in EM. As shown in Supplementary Fig. S2b, xenograft tumors derived from IHCC organoids after hepatocyte differentiation were CK19-positive adenocarcinomas with glandular and tubular structures, being compatible with IHCC, and not HCC. On the other hand, hepatocyte markers such as HNF4A and albumin were also positive (Supplementary Fig. S2b). Since xenograft tumors grow under the skin of SCID mice, factors involved in induction to IHCC, such as R-spondin 1, may be present in the serum and/or stromal cells derived from SCID mice, possibly inhibiting the hepatocyte differentiation of IHCC organoids. These *in vitro* and *in vivo* studies indicated that hepatocyte differentiation upon culture in DM reduced the malignant potential of IHCC organoids.

**Figure 5:**
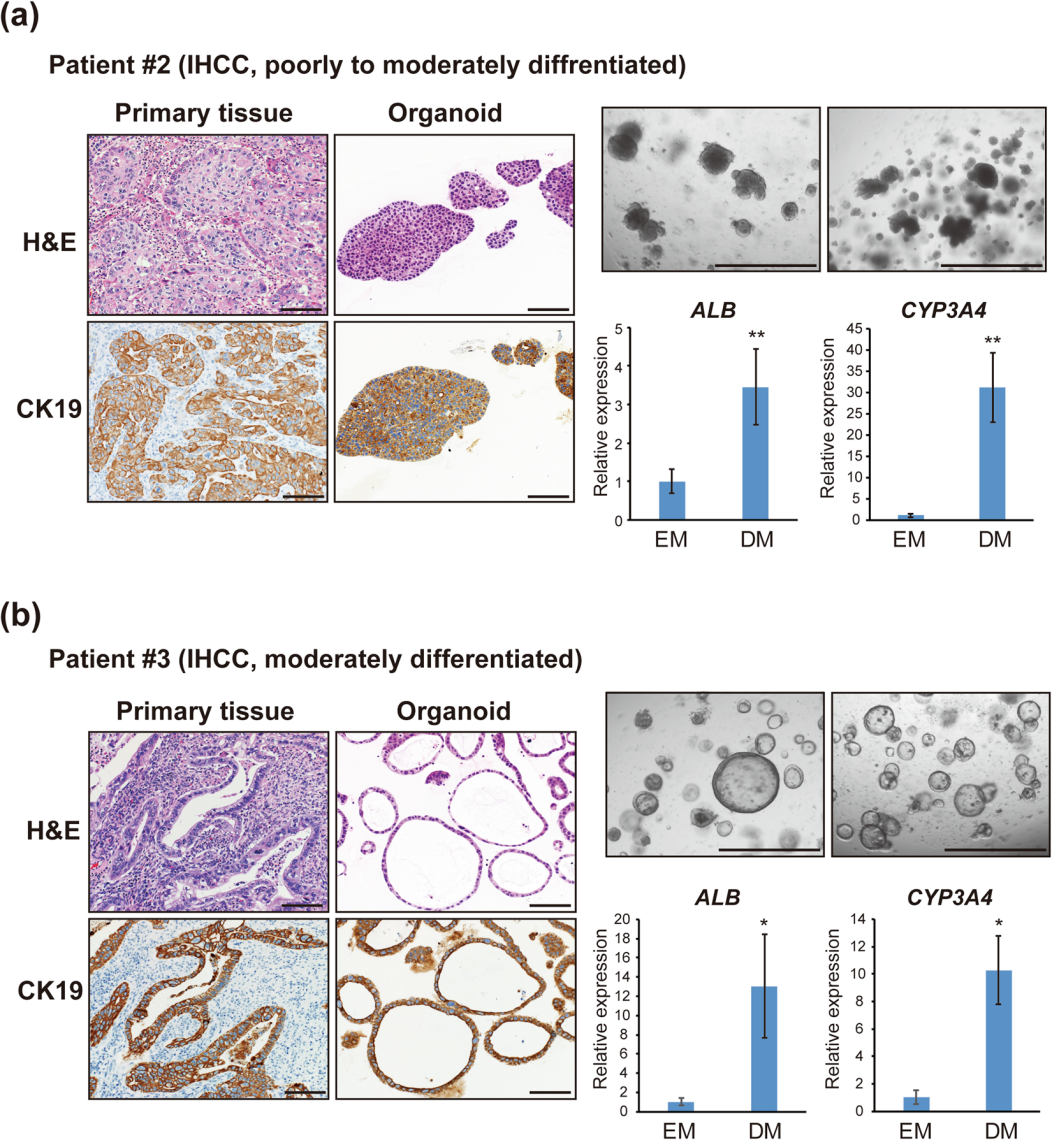
Induction of hepatocyte differentiation of organoids derived from surgically resected IHCC tissues. Organoids were established using surgically resected tissues of poorly to moderately differentiated IHCC from patient #2 (**a**) and moderately differentiated IHCC from patient #3 (**b**). H&E and CK19 staining of the primary tissues and organoids derived from IHCC cases (scale bars: 100 μm) and bright-field images of IHCC organoids (scale bars: 1000 μm) are shown. After stable culture over 10 passages, these IHCC organoids were subjected to hepatocyte differentiation using the same protocol, and the relative expression of *Albumin* (*ALB*) and *CYP3A4* in organoids that had been cultured in EM or DM were examined.

To further validate hepatocyte differentiation in IHCC organoids, we established two organoids from cancer tissues that had been surgically resected from patients with IHCC. As shown in Fig. 5, a second set of organoids were established from surgically resected samples of poorly to moderately differentiated IHCC derived from patient #2 (an 80-year-old male), and a third set of organoids were established from surgically resected samples of moderately differentiated IHCC derived from patient #3 (a 46-year-old male). As shown in Fig. 5a, histopathological examination of H&E-stained primary IHCC tissue (patient #2) with CK19 immunostaining demonstrated poorly to moderately differentiated adenocarcinoma with a solid growth pattern. H&E and CK19 staining of organoids derived from this IHCC case also demonstrated a solid structure. On the other hand, histopathological examination of the primary IHCC tissue (patient #3) demonstrated moderately differentiated adenocarcinoma with glandular and tubular structures. Organoids derived from this IHCC case also formed a monolayered cystic structure, with a morphology similar to that of IHCC organoids derived from moderately differentiated IHCC #1 (Figs 1c and 5b). Thus, these morphologic features of IHCC organoids highly recapitulated the histopathological features of the primary IHCCs, including tumor differentiation (Figs 1c and 5a,b). These results indicate that IHCC organoids retain the histopathological structures of the primary tumors. Recent studies have also reported histopathological similarity between organoids and primary tissues for prostate, pancreatic and colon cancers^23,24,26^. After stable culture over 10 passages, these IHCC organoids were subjected to hepatocyte differentiation using the same protocol. As shown in Fig. 5a and b, *Albumin* and *CYP3A4*, markers of mature hepatocytes, were markedly upregulated after hepatocyte differentiation of IHCC organoids.

**Figure 6:**
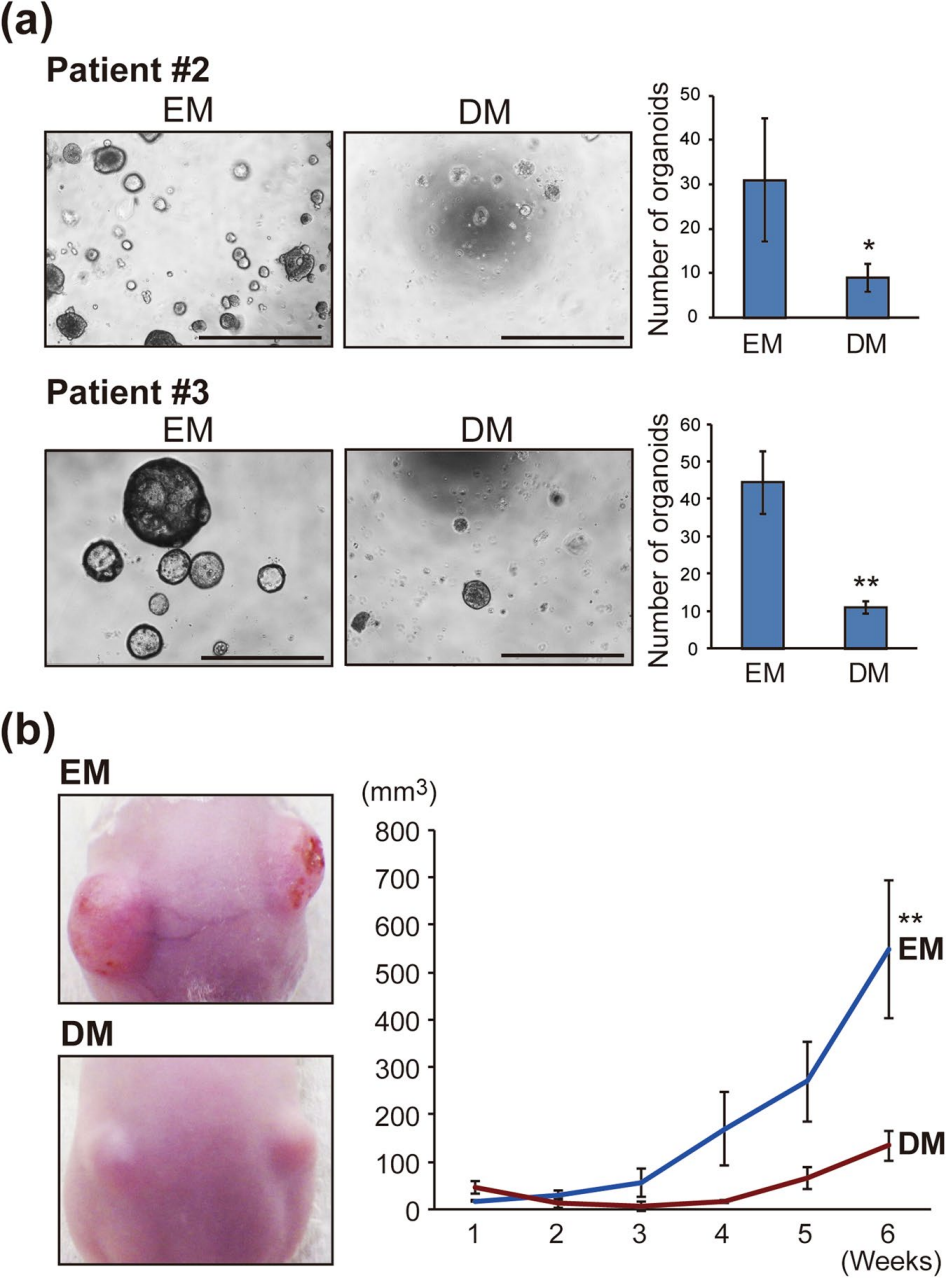
Hepatocyte differentiation reduces the malignant potential of organoids derived from surgically resected IHCC tissues. (**a**) Number of IHCC organoids derived from patients #2 and #3 cultured in EM or DM. IHCC organoids were cultured in EM or DM for 12 days, then cultured in EM for 10 days. The numbers of organoids were counted. Scale bars: 500 μl. (**b**) Tumor volumes of xenografted IHCC organoids derived from patient #3 cultured in EM or DM. We implanted 1 × 10^6^ cells of IHCC organoids cultured in EM or DM subcutaneously into the backs of SCID mice. Tumor volumes of xenografted IHCC organoids cultured in EM (n = 4) or DM (n = 4) on SCID mice were measured.

We investigated the malignant potential of IHCC organoids derived from patients #2 and #3 after hepatocyte differentiation i*n vitro* and *in vivo*. The numbers of IHCC organoids derived from patients #2 and #3 were markedly decreased after culture in DM (Fig. 6a). IHCC organoids derived from patient #3 cultured in EM or DM (1 × 10^6^ cells) were implanted subcutaneously into the backs of SCID mice. As shown Fig. 6b, the volumes of xenografted tumors of IHCC organoids cultured in DM (n = 4) were significantly reduced in comparison to those of IHCC organoids cultured in EM (n = 4). We also implanted IHCC organoids derived from patient #2 into SCID mice, but the organoids did not form tumors. As we confirmed that IHCC organoids derived from patient #2 harbor the driver gene mutations including *TP53* and *IDH1*, and can be stably cultured for over one year, we consider these organoids to be a cancer organoid line, although they did not form tumors on SCID mice.

### Wnt3a derived from recruited macrophages may convert hepatocytes to biliary lineage cells

Our results indicated that inhibition of Wnt signaling by removal of R-spondin 1 is critical for hepatocyte differentiation of IHCC organoids, paradoxically suggesting that activation of Wnt signaling may play critical roles in the initiation and progression of IHCC. Boulter *et al*. have revealed that macrophage-derived Wnt3a promotes hepatocyte regeneration in chronic liver injury^30^. Therefore, we hypothesized that excessive secretion of Wnt3a from macrophages might induce malignant transformation of mature hepatocytes to IHCC cells.

**Figure 7:**
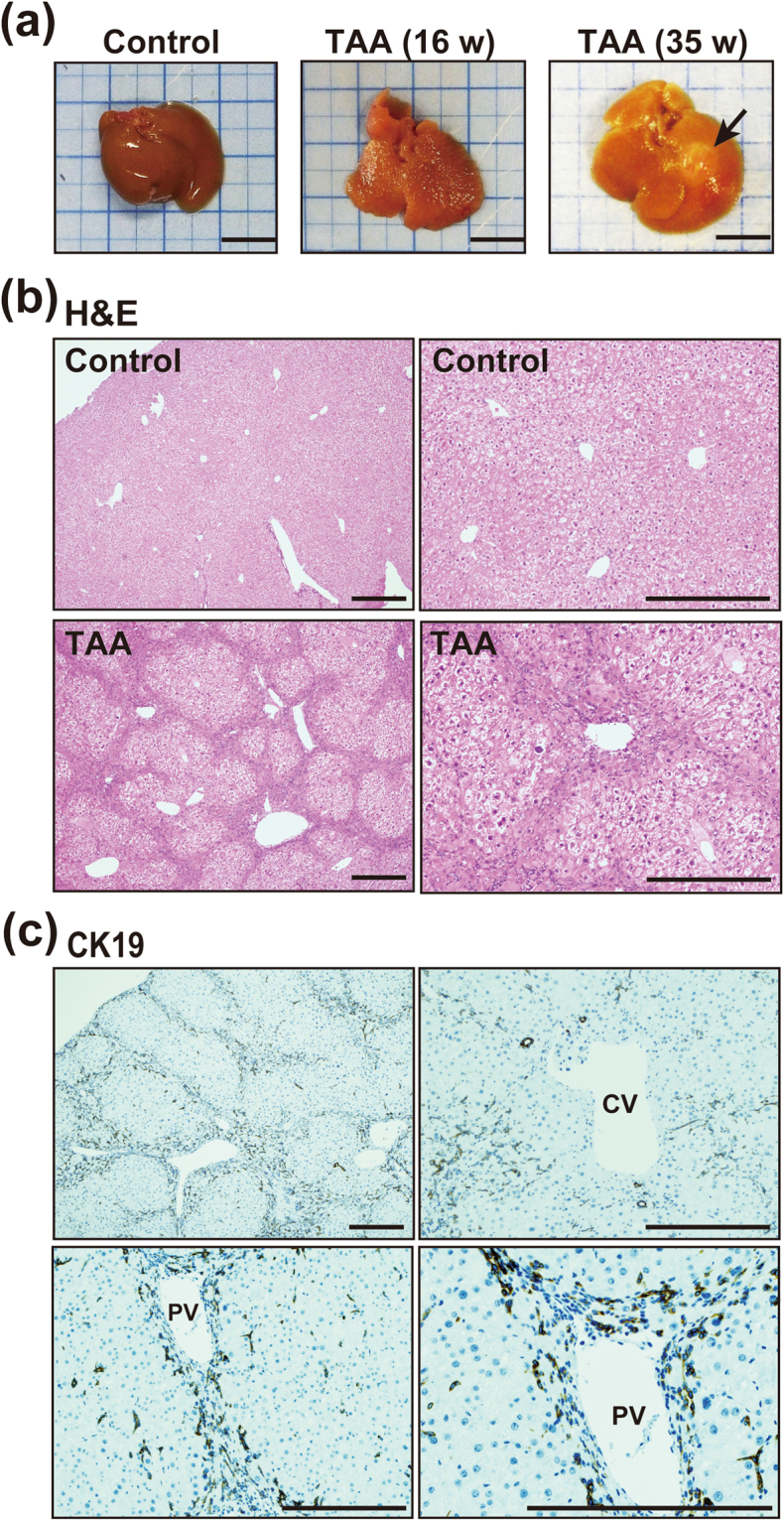
TAA-induced liver injury may promote conversion of hepatocytes to biliary lineage cells. (**a**) Macroscopic appearance of the liver of mice administered TAA for 16 and 35 weeks. Administration of TAA induced prominent inflammation in the liver by16 weeks and development of liver tumors (arrow) by 35 weeks. Scale bars: 10 mm. (**b**) H&E staining of the liver of control mice and mice administered TAA for 16 weeks. Administration of TAA induced prominent inflammation and fibrosis around Glisson’s capsule in the liver by 16 weeks and development of liver tumors by 35 weeks. Scale bars: 300 μm. (**c**) Immunohistochemical staining of CK19 in the liver of control mice and mice administered TAA for 16 weeks. The cholangiocyte marker CK19 was strongly expressed in cells around portal veins (PV) in the liver, whereas cells around central veins (CV) showed no expression of CK19. Scale bars: 300 μm.

To investigate the role of macrophages in the development of IHCC cells, we used a mouse model of IHCC generated by administration of TAA^12,31^. TAA induced prominent inflammation and fibrosis around Glisson’s capsule in the liver by 16 weeks and liver tumors developed by 35 weeks (Fig. 7a and b). At 16 weeks, the cholangiocyte marker CK19 was strongly expressed in cells around the portal veins in the liver, whereas cells around central veins showed no expression of CK19 (Fig. 7c). These findings suggested that periportal hepatocytes may be converted to biliary lineage cells in response to TAA-induced liver injury.

**Figure 8:**
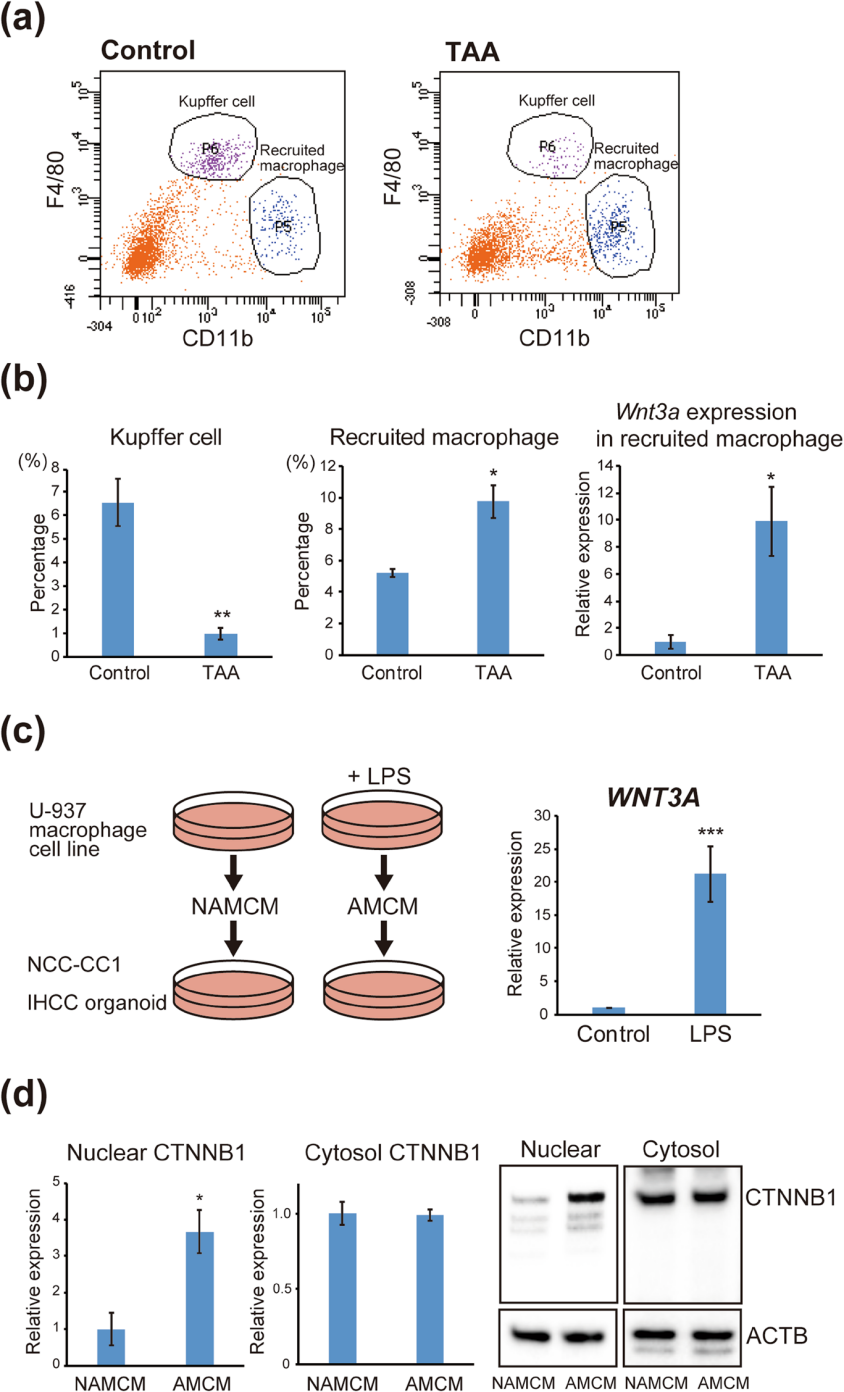
Activation of the Wnt signaling pathway by macrophages recruited to the liver by inflammation. (**a**) Flow cytometry analysis to determine the proportions of resident macrophages (Kupffer cells) and macrophages recruited to the liver of TAA-administered mice. Kupffer cells were isolated as F4/80^high^ and CD11b^low^ cells, and recruited macrophages as F4/80^low^ and CD11b^high^ cells. (**b**) The percentages of Kupffer cells and recruited macrophages in the liver of control mice and TAA-administered mice (left and middle). Relative expression of *Wnt3a* derived from recruited macrophages in the liver of control mice and TAA-administered mice (right). (**c**) Scheme of the LPS stimulation assay (left). U937 human macrophage cells were activated by treatment with 5 μg/ml LPS for 48 h. U-937 cells treated with PBS were used as a negative control. Activated macrophage conditioned medium (AMCM) and non-activated macrophage conditioned medium (NAMCM) were collected. The NCC-CC1 IHCC cells and IHCC organoids were then treated with AMCM or NAMCM for 48 hours. Relative expression of *WNT3A* in the U937 human macrophage cell line untreated or treated with LPS was examined (right). (**d**) Western blot analysis of nuclear and cytosolic β-catenin (CTNNB1) in NCC-CC1 cells (left) and IHCC organoids (right) treated with NAMCM or AMCM. β-Actin (ACTB) was used as an internal control.

Macrophages are classified as resident macrophages (also referred to as Kupffer cells or tissue macrophages), and recruited macrophages are derived from bone marrow^32,33^. Using flow cytometry analysis, we examined the proportions of resident macrophages (Kupffer cells) and recruited macrophages in the liver of TAA-administered mice. As shown in Fig. 8a, Kupffer cells were isolated as F4/80^high^ and CD11b^low^ cells, and recruited macrophages as F4/80^low^ and CD11b^high^ cells^32^. We found that TAA-induced inflammation in the liver resulted in a prominent decrease of Kupffer cells and an increase of recruited macrophages (Fig. 8b). We extracted mRNA from the recruited macrophages and examined the expression of *Wnt3a*. As shown in Fig. 8b, *Wnt3a* expression was markedly increased in these recruited macrophages after TAA administration. These findings suggested that liver inflammation induces recruited macrophages to secrete Wnt3a, and that this macrophage-derived Wnt3a may convert mature hepatocytes into biliary lineage cells.

### Activation of macrophages by LPS stimulation induces nuclear translocation of β-catenin in IHCC cells

We next examined the relationship between macrophage-derived WNT3A and nuclear translocation of β-catenin in IHCC cells.

We treated the U937 human macrophage cells with 5 μg/ml lipopolysaccharide (LPS) for 48 hours to activate the macrophages. Expression of *WNT3A* was significantly increased in these macrophages upon LPS stimulation (Fig. 8c). Activated macrophage conditioned medium (AMCM) and non-activated macrophage conditioned medium (NAMCM) were collected. The NCC-CC1 IHCC cells and IHCC organoids were then treated with AMCM or NAMCM for 48 hours (Fig. 8c). Western blot analysis showed that the nuclear β-catenin protein level was significantly increased in IHCC cells and organoids treated with AMCM, although there was no difference in the levels of cytosol β-catenin protein between IHCC cells and organoids treated with AMCM and NAMCM (Fig. 8d). These results suggested that macrophage-derived WNT3A induced nuclear translocation of β-catenin, possibly promoting the malignant transformation of mature hepatocytes into IHCC cells.

## Discussion

Here we have described the successful establishment and long-term *in vitro* culture of organoids derived from human IHCC tissues. Since organoids closely recapitulate the properties of primary tumors^25,26^, IHCC organoids can be a powerful research tool for clarifying the mechanisms underlying the initiation of IHCC and for development of novel therapies.

Recent advances in cell biology have revealed that mature hepatocytes can be converted to bile duct cells^13,14^. Conversely, adult bile duct-derived progenitor cells can be converted to functional hepatocytes^27^. These findings suggest that mutual phenotypic plasticity exists between mature hepatocytes and bile duct cells. In the present study, we have demonstrated that IHCC cells can reacquire functions of mature hepatocytes, such as secretion of albumin and bile acid and CYP3A4 activity, even after malignant transformation. Moreover, the malignant potential of IHCC cells was markedly reduced after hepatocyte differentiation. Removal of R-spondin 1, the ligand of LGR5, induced differentiation of IHCC cells into functional hepatocytes, suggesting that inhibition of the Wnt signaling pathway may induce hepatocyte differentiation.

Studies using a mouse model of IHCC have indicated that the Notch signaling pathway is important for conversion of hepatocytes to IHCC cells^11,12^. Therefore, we investigated the effect of the Notch signaling pathway on hepatocyte differentiation of IHCC organoids by excluding DAPT from DM (Supplementary Fig. S1a and b). DAPT is a γ-secretase inhibitor that indirectly inhibits the Notch signaling pathway. Our results indicate that DAPT is important for suppression of genes associated with stemness and EMT, but has less effect on hepatocyte differentiation of IHCC organoids, suggesting that inhibition of Notch signaling is important for reduction of the malignant potential of human IHCC organoids.

Huch *et al*. have demonstrated that normal bile duct-derived organoids can be converted to functional hepatocytes *in vitro* and upon transplantation *in vivo*. Expression of hepatocyte markers such as albumin and CYP3A4 after hepatocyte differentiation was higher in normal bile duct-derived organoids than in the IHCC organoids investigated in our study. Recent studies have revealed that IHCC harbors various gene mutations, including *TP53*, *KRAS*, *ARID1A*, *IDH1*, *MCL1* and *PBRM1*^34^. In addition, Saha *et al*. have shown that mutant IDH inhibits HNF4a to block hepatocyte differentiation and promote biliary cancer^35^. Genetic and epigenetic alterations in IHCC organoids may inhibit hepatocyte differentiation in comparison with normal bile duct-derived organoids, which may reflect the discrepancy in hepatocyte differentiation between IHCC and the normal bile duct.

Hepatocyte differentiation was conducted only by conversion of the culture medium from EM to DM. Expression of DNA methyltransferases was decreased by removal of R-spondin 1 from EM. In addition, treatment of IHCC organoids with the DNA demethylating agent 5-Aza-CdR increased the expression of markers for mature hepatocytes. These findings suggest that hepatocyte differentiation is modulated by epigenetic alterations and that inhibition of DNA methylation induces differentiation of IHCC cells to mature hepatocytes. However, during hepatocyte differentiation we found no significant difference in DNA methylation levels around the promoter regions of genes associated with mature hepatocytes, including *HNF4A*. Further studies will be necessary to clarify the molecular mechanism(s) underlying the differentiation of IHCC cells to mature hepatocytes.

We have demonstrated that inhibition of Wnt signaling is important for hepatocyte differentiation, paradoxically suggesting that activation of Wnt signaling plays critical roles in the transdifferentiation of mature hepatocytes to bile duct cells and the initiation and progression of IHCC. Our results suggest that recruited macrophages activate the Wnt signaling pathway and that Wnt3a derived from these macrophages promotes malignant transformation of mature hepatocytes to IHCC cells. Touboul *et al*. have reported that stage-specific regulation of the Wnt/β-catenin pathway enhances differentiation of human embryonic stem cells to hepatocytes^36^. In addition, several studies have shown that Wnt/β-catenin signaling is activated in cholangiocarcinoma^37–39^. These findings might be relevant to our results showing that Wnt3a derived from macrophages recruited to the liver by inflammation contributes to transdifferentiation of mature hepatocytes into biliary lineage cells and the initiation of IHCC. Further *in vivo* lineage tracing studies will be needed to support this hypothesis.

In conclusion, our study using organoid models of human IHCC has indicated that IHCC cells can reacquire functions of mature hepatocytes by inhibition of Wnt signaling. On the other hand, Wnt3a derived from macrophages recruited to the liver by inflammation may promote the malignant transformation of mature hepatocytes to IHCC cells. The results of the present study support the recently proposed hypothesis that IHCC cells are derived from hepatocytes. As modulation of the Wnt signaling pathway appears to be critical for transdifferentiation between mature hepatocytes and IHCC cells, inhibition of this pathway might be a promising approach for prevention of IHCC, especially in patients with chronic inflammatory liver diseases such as viral hepatitis.

## Methods

### Establishment of xenografts

IHCC tissues were cut into small pieces (2–4 mm^3^ fragments) and then implanted subcutaneously into 5–7-week-old congenitally athymic female C.B17/Icr-scid (scid/scid) mice (CLEA Japan, Tokyo, Japan), as described previously^28^. SCID mice were bred and housed under specific pathogen-free conditions at the Animal Center of Keio University Faculty of Pharmacy. All experiments and procedures were approved by the Keio University Animal Research Committee, and all methods were carried out in accordance with the approved guidelines. Figure 1a shows the macroscopic appearance of the IHCC xenograft tumor that had been implanted subcutaneously into a SCID mouse for approximately 3 months. The xenograft tumor excised from the SCID mouse was subsequently subjected to organoid culture.

### Organoid culture

For organoid culture we employed xenograft tissues^28^ and specimens surgically resected from IHCC patients at the National Cancer Center Hospital (Tokyo, Japan). This study was approved by the Ethics Committees of the National Cancer Center and Keio University (Tokyo, Japan), and all methods were carried out in accordance with the approved guidelines. Written informed consent was obtained from all of the patients.

The xenograft tumor tissues and the surgically resected tumor tissues were cut into small pieces and incubated in digestion buffer for 1 hour at 37 °C. The digestion buffer was composed of Dulbecco’s modified Eagle medium (DMEM) with 2.5% fetal bovine serum, 0.0125% dispase type II (Thermo Fisher Scientific) and 0.0125% collagenase type XI (Sigma-Aldrich). Fragments were allowed to settle under normal gravity for 1 minute, and the supernatant was collected in a tube and centrifuged at 800 rpm for 5 minutes. The pellet was washed with PBS and centrifuged at 800 rpm for 5 minutes twice. Isolated cells were embedded in Matrigel (growth factor reduced, phenol red-free; Corning) on ice and seeded in 48-well plates.

The expansion medium (EM) was composed of Advanced DMEM/F12 (Thermo Fisher Scientific) supplemented with Glutamax, 10 mM HEPES, penicillin/streptomycin, 1 × N2 supplement, 1 × B27 supplement, 50 ng/mL EGF (all from Thermo Fisher Scientific), 1.25 mM N-acetylcysteine, 50 nM gastrin, 10 mM nicotinamide (all from Sigma-Aldrich) and R-spondin 1 (10% conditioned medium from R-spondin 1-producing cell lines). For culture of organoids derived from IHCC patients #2 and #3, 5 μM A83-01 (Tocris) and 10 μM forskolin (Tocris) were added.

### Hepatocyte differentiation

As shown in Fig. 2a, hepatocyte differentiation using IHCC organoids was conducted as described previously^27^. IHCC organoids were cultured for 7–10 days in EM additionally supplemented with BMP7 (25 ng/ml). Then, the culture medium was changed to differentiation medium (DM) composed of Advanced DMEM/F12 supplemented with Glutamax, 10 mM HEPES, penicillin/streptomycin, 1 × N2 supplement, 1 × B27 supplement, 50 ng/mL EGF, 50 nM gastrin, 25 ng/ml HGF (Peprotech), 100 ng/ml FGF19 (R&D systems), 10 μM DAPT (Sigma-Aldrich), 25 ng/ml BMP7 (Peprotech), and 30 μM dexamethasone (Sigma-Aldrich). DM was changed every 2–3 days for a period of 12 days.

### Microarray analysis

Total RNAs were extracted from organoids using the RNeasy Mini kit (Qiagen). Microarray analysis of total genes was conducted by Toray Industries. In brief, extracted total RNA was checked with a Bioanalyzer (Agilent Technologies) and labeled with Cy5 and Cy3. The labeled RNAs were hybridized onto a Human Oligo chip 25k (Toray Industries). After stringent washing, the fluorescent signals were scanned with a 3D-Gene Scanner (Toray Industries) and analyzed using the 3D-Gene Extraction software (Toray Industries). The raw data for each spot were normalized by subtraction of the mean background signal intensity determined from the signal intensities of all blank spots with 95% confidence intervals. The relative expression level was calculated by comparing the signal intensities of the valid spots throughout the microarray experiments. All data were submitted to the GEO database under the accession number GSE93908.

### Gene set enrichment analysis (GSEA)

GSEA was performed using the database from version 3.1 of the molecular signature database: the C2 curated gene sets from online pathway databases, PubMed publications and knowledge of domain experts.

The HOSHIDA_LIVER_CANCER_SUBCLASS_S3 gene set (genes from the ‘subtype S3’ signature of hepatocellular carcinoma: hepatocyte differentiation) was used for GSEA^29^.

### Immunohistochemical staining

Sections of formalin-fixed, paraffin-embedded organoids and tissues were deparaffinized and rehydrated. For antigen retrieval, the sections were heat-treated in an autoclave for 20 minutes at 121 °C in 10 mM citrate buffer (pH 6.0). The sections were then incubated with antibodies against KI67 (Dako) and cytokeratin 19 (CK19) (ab52625, Abcam).

### Western blotting

Protein extracts were separated by SDS/polyacrylamide gel electrophoresis and transferred to nitrocellulose membranes (GE Healthcare Life Sciences). The membranes were then hybridized with antibodies against CK19 (ab7754, Abcam), CD44 (15675-1-AP, Proteintech), CD133 (W6B3C1, Miltenyi Biotec) and LGR5 (ab75732, Abcam). β-Actin (sc-47778, Santa Cruz Biotechnology) was used as an internal control.

### Immunofluorescence staining

Organoids were cultured on chamber slides for 3 days and then fixed with 4% paraformaldehyde (PFA). Immunofluorescence staining was performed with the mouse anti-CK19 monoclonal antibody (ab7754, Abcam). The Alexa 568-conjugated anti-mouse antibody was used as the secondary antibody. Sections of formalin-fixed, paraffin-embedded organoids were deparaffinized and rehydrated. For antigen retrieval, the sections were treated in 10 mM citrate buffer (pH 6.0) for 20 minutes at 121 °C in an autoclave. Images of the organoids were obtained using confocal microscopy (FV1000, Olympus).

### Quantitative RT-PCR

Total RNAs were extracted from organoids using the RNeasy Mini kit (Qiagen) and cDNAs were synthesized using Multiscribe Reverse Transcriptase (Thermo Fisher Scientific). Quantitative RT-PCR was performed using the Universal SYBR Select Master Mix (Thermo Fisher Scientific) in accordance with the manufacturer’s instructions. Quantitative analyses were performed using the CFX96 Real-Time System (BioRad). The primers are listed in Supplementary Table S1. GAPDH was used as an internal control. All experiments were carried out in triplicate.

### Measurement of albumin and bile acid secretion

To assess the ability of organoids to secrete albumin, culture medium was collected 48 hours after the last medium change. Albumin in the collected supernatant was quantified by Western blotting using an antibody against human serum albumin (ab8940; Abcam). To assess the ability of organoids to secrete bile acid, culture medium was collected 48 hours after the last medium change. Bile acid in the collected supernatant was quantified using Total Bile Acids Test Wako (Wako Pure Chemical Industries) in accordance with the manufacturer’s instructions.

### CYP3A4 activity

To measure CYP3A4 activity, cells were removed from Matrigel and cultured with the Luciferin-PFBE substrate (50 μM) in Advanced DMEM/F12 medium supplement with 10% FBS (Thermo Fisher Scientific). Cytochrome P450 activity was measured 8 hours later using the P450-Glo Assay Kit (Promega) in accordance with the manufacturer’s instructions.

### Sphere formation assay and organoid counting

For the sphere formation assay, organoids cultured in EM or DM were reseeded at 1.0 × 10^3^ cells in ultra-low-attachment plates (Corning) and then cultured in EM or Advanced DMEM/F12 with 10% FBS for 10 days. For the organoid counting assay, organoids cultured in EM or DM were reseeded at 1.0 × 10^3^ cells in Matrigel and then cultured in EM or Advanced DMEM/F12 with 10% FBS for 10 days. The numbers of spheres and organoids were counted.

### Tumor formation assay

We implanted 1 × 10^6^ cells of IHCC organoids cultured in EM or DM subcutaneously into the backs of SCID mice. Tumor volumes of xenografted IHCC organoids cultured in EM (n = 4–8) or DM (n = 4–8) on SCID mice were measured.

### Mouse model of IHCC

A mouse model of IHCC was prepared as described previously^12,31^. To induce IHCC, C57BL/6 mice were allowed access to drinking water containing 300 mg/L thioacetamide (TAA, Wako Pure Chemical Industries) for 35 weeks. We used 19 C57BL/6 mice as control mice (n = 8) and TAA-treated mice (n = 11). All experiments and procedures were approved by the Keio University Animal Research Committee, and all methods were carried out in accordance with the approved guidelines.

### Macrophage isolation

For tissue isolation, mice were killed and the livers were removed. Liver-resident and recruited macrophages were separated as described previously^40^. Briefly, the livers were minced and filtered without any enzymatic digestion. The constituent cells were resuspended in 25% Percoll solution (GE Healthcare Life Science) and gradient-centrifuged with 50% Percoll solution. After blocking with an anti-FcR antibody (CD16/32; BD Pharmingen, San Diego, CA) for 30 minutes at 4 °C, the cells were incubated with specific fluorescence-labeled monoclonal antibodies at 4 °C for 30 minutes. For sorting of liver-resident and recruited macrophages, anti-F4/80 (eBioscience), anti-7-AAD (eBioscience), anti-CD45.2 (BD Pharmingen) and anti-CD11b (BD Pharmingen) monoclonal antibodies were used.

### Cell lines and LPS stimulation assay

The macrophage cell line U-937 was purchased from the JCRB cell bank (Osaka, Japan), and the IHCC cell line NCC-CC1 was provided by the National Cancer Center (Tokyo, Japan)^28^. The U-937 and NCC-CC1 cells were cultured in RPMI1640 medium supplemented with 10% FBS. The U-937 macrophage cells were treated with 5 μg/ml LPS for 48 hours. U-937 cells treated with PBS were used as a negative control. Activated macrophage conditioned medium (AMCM) and non-activated macrophage conditioned medium (NAMCM) were collected. The NCC-CC1 IHCC cells and IHCC organoids were then treated with AMCM or NAMCM for 48 hours (Fig. 8c).

Extraction of nuclear proteins and cytoplasmic proteins was performed using the NE-PER Nuclear and Cytoplasmic Extraction Reagent Kit (Thermo Fisher Scientific) in accordance with the manufacturer’s instructions. Protein extracts were separated by SDS/polyacrylamide gel electrophoresis and transferred to nitrocellulose membranes (GE Healthcare Life Sciences). The membranes were then hybridized with antibody against β-catenin (sc-7199, Santa Cruz Biotechnology). β-Actin (sc-47778, Santa Cruz Biotechnology) and histone H3 (sc-8654, Santa Cruz Biotechnology) were used as internal controls.

### DNA methylation assay

Genome-wide analysis of DNA methylation using BeadChip assay with Infinium HumanMethylation450 (Illumina) was conducted by Takara. For the DNA demethylation assay, organoids were treated with 1 μM 5-aza-2’-deoxycytidine (5-Aza-CdR, Sigma-Aldrich). After 24 hours, the medium containing 5-Aza-CdR was replaced with regular culture medium.

### Statistical analysis

Three or four independent experiments were performed for all studies. All statistical analyses were performed using R studio. Student’s *t* test, and Dunnett’s test were employed (*p < 0.05, **p < 0.01, ***p < 0.001. All error bars represent standard deviation).

## Electronic supplementary material

Supplementary Information
