# Supplementary material for: Induction of differentiation of intrahepatic cholangiocarcinoma cells to functional hepatocytes using an organoid culture system
Source: Sci Rep. 2018 Feb 12;8:2821. doi: 10.1038/s41598-018-21121-6 (PMC5809480; doi:10.1038/s41598-018-21121-6)
Supplement: Supplementary file 1 — Supplementary Information [file 41598_2018_21121_MOESM1_ESM.docx]

**Supplementary Information**

**Induction of differentiation of intrahepatic cholangiocarcinoma cells**

**to functional hepatocytes using an organoid culture system**

Yoshimasa Saito, Toshiaki Nakaoka, Toshihide Muramatsu, Hidenori Ojima,

Aoi Sukeda, Yuko Sugiyama, Ryoei Uchida, Ryo Furukawa, Aya Kitahara,

Toshiro Sato, Yae Kanai and Hidetsugu Saito

**Figure legends**

**Supplementary Fig. S1**

(a) Relative expression of *Albumin (ALB)*, *CYP3A4* and *HNF4A* in IHCC organoids cultured in EM or DM. One specific factor, such as BMP7, FGF19, DAPT or dexamethasone, was excluded from DM.

(b) Relative expression of *CD44*, *Snail1 (SNAI1)*, *Vimentin* *(VIM)* and *E-cadherin (CDH1)* in IHCC organoids cultured in EM or DM. One specific factor, such as BMP7, FGF19, DAPT or dexamethasone, was excluded from DM.

**Supplementary Fig. S2**

(a) Relative expression of *Snail1 (SNAI1)*, *Vimentin (VIM)* and *E-cadherin* *(CDH1)* in IHCC organoids cultured in EM or DM.

(b) H&E, CK19, HNF4A and Albumin (ALB) staining of xenograft tumors derived from IHCC organoids after hepatocyte differentiation. Scale bars: 100 μm.

| **Supplementary Table S1: Primer sequences for RT-PCR** | | |
| --- | --- | --- |
|  |  |  |
| Gene name | Primer sequence | |
|  | Forward (5' to 3') | Reverse (5' to 3') |
| *ALB* | CTGCCTGCCTGTTGCCAAAGC | GGCAAGGTCCGCCCTGTCATC |
| *HNF4a* | ATTGACAACCTGTTGCAGGA | CGTTGGTTCCCATATGTTCC |
| *CYP3A4* | TTCAGCAAGAAGAACAAGGACAA | GGTTGAAGAAGTCCTCCTAAGC |
| *CD44* | TGGCACCCGCTATGTCCAG | GTAGCAGGGATTCTGTCTG |
| *DNMT1* | AGAACGGTGCTCATGCTTACA | CTCTACGGGCTTCACTTCTTG |
| *DNMT3B* | CCCAGCTCTTACCTTACCATCG | GGTCCCCTATTCCAAACTCCT |
| *WNT3A* | CCTGCACTCCATCCAGCTACA | GACCTCTCTTCCTACCTTTCCCTTA |
| *Vimentin* | AACGCCAGATGCGTGAAATG | CCAGAGGGAGTGAATCCAGATTA |
| *E-cadherin* | TACGCCTGGGACTCCA | CCAGAAACGGAGGCCT |
| *Snail1* | AAGGCCTTCTCTAGGCCCT | GCAGGTTGGAGCGGTCAG |
| *Wnt3a* (mouse) | CTCCTCTCGGATACCTCTTAGTG | CCAAGGACCACCAGATCGG |
| *GAPDH* | CACCACCATGGAGAAG | GCTAAGCAGTTGGTGG |

| **Supplementary Table S2** | | | | | | | |  |  |
| --- | --- | --- | --- | --- | --- | --- | --- | --- | --- |
| **The result of BeadChip assay: AVG_Beta (EM) > 0.3 and AVG_Beta Ratio (EM/DM) > 2.0** | | | | | | | | | |
| TargetID | AVG_Beta (EM) | Intensity (EM) | AVG_Beta (DM) | Intensity (DM) | CHR | UCSC_REFGENE_  NAME | UCSC_REFGENE_  ACCESSION | CPG_  ISLAND | Ratio (EM/DM) |
| cg05301609 | 0.41278 | 70 | 0.00227 | 305 | 17 | ALDH3A2 | NM_000382 |  | 181.8 |
| cg03441493 | 0.33371 | 50 | 0.00722 | 27 | Y |  |  | Island | 46.2 |
| cg15752756 | 0.32833 | 344 | 0.02546 | 116 | 6 | HLA-DQB1 | NM_002123 | S_Shore | 12.9 |
| cg23241694 | 0.31933 | 205 | 0.0268 | 3 | 17 |  |  | N_Shore | 11.9 |
| cg05051262 | 0.3476 | 393 | 0.03277 | 208 | Y |  |  | Island | 10.6 |
| cg27020349 | 0.41617 | 282 | 0.04239 | 164 | 6 | UTRN | NM_007124 |  | 9.8 |
| ch.5.706755F | 0.34803 | 9269 | 0.03815 | 4110 | 5 | ZFR | NM_016107 |  | 9.1 |
| cg02050847 | 0.41527 | 112 | 0.04652 | 58 | Y | RPS4Y2 | NM_001039567 |  | 8.9 |
| cg10996368 | 0.53651 | 13064 | 0.06535 | 7849 | 11 | NTM | NM_001144059 |  | 8.2 |
| cg14467015 | 0.30138 | 518 | 0.04279 | 393 | Y | TTTY13 | NR_001537 |  | 7.0 |
| cg00272582 | 0.55362 | 197 | 0.08478 | 73 | Y | ZFY | NM_003411 | Island | 6.5 |
| cg02011394 | 0.36244 | 57 | 0.05722 | 52 | Y | TSPY4 | NM_001164471 | Island | 6.3 |
| ch.10.109847071F | 0.42832 | 9382 | 0.07639 | 9428 | 10 |  |  |  | 5.6 |
| cg23614811 | 0.50477 | 102 | 0.0917 | 10 | 17 |  |  |  | 5.5 |
| cg06578800 | 0.69797 | 231 | 0.14228 | 35 | 17 |  |  | S_Shore | 4.9 |
| cg05785424 | 0.49398 | 208 | 0.10487 | 173 | 17 |  |  |  | 4.7 |
| cg22648282 | 0.31773 | 47 | 0.0684 | 7 | 17 | C17orf51 | NM_001113434 | N_Shore | 4.6 |
| cg03791497 | 0.35585 | 88 | 0.07961 | 61 | 17 | TMEM11 | NR_024547 | S_Shore | 4.5 |
| cg21767373 | 0.51356 | 106 | 0.11774 | 164 | 17 | DHRS7B | NM_015510 | Island | 4.4 |
| cg06109624 | 0.46269 | 86 | 0.10801 | 27 | X | MCF2 | NM_005369 |  | 4.3 |
| cg15416179 | 0.38226 | 88 | 0.09061 | 24 | 17 | MAP2K3 | NM_145109 | S_Shore | 4.2 |
| cg02875834 | 0.6333 | 177 | 0.15241 | 93 | X | DIAPH2 | NM_006729 | Island | 4.2 |
| cg16833444 | 0.31481 | 656 | 0.07707 | 352 | 17 |  |  |  | 4.1 |
| cg02402208 | 0.3819 | 310 | 0.09546 | 342 | Y |  |  | N_Shore | 4.0 |
| cg10620659 | 0.58693 | 249 | 0.14811 | 166 | Y | EIF1AY | NM_004681 | N_Shore | 4.0 |
| cg02129146 | 0.34896 | 54 | 0.0917 | 10 | Y |  |  | N_Shelf | 3.8 |
| cg10441661 | 0.30524 | 44 | 0.08332 | 32 | 17 |  |  | S_Shore | 3.7 |
| cg16248432 | 0.60319 | 152 | 0.17042 | 315 | 12 | DIP2B | NM_173602 |  | 3.5 |
| cg04294990 | 0.36317 | 270 | 0.10659 | 12 | 17 | USP22 | NM_015276 | N_Shore | 3.4 |
| cg04630982 | 0.48847 | 121 | 0.14506 | 140 | X | MIR505 | NR_030230 |  | 3.4 |
| cg11747279 | 0.43325 | 76 | 0.13015 | 62 | 17 |  |  |  | 3.3 |
| cg11413133 | 0.53701 | 116 | 0.16159 | 19 | 17 | KCNJ12 | NM_021012 | Island | 3.3 |
| cg13557397 | 0.46521 | 87 | 0.14158 | 101 | 17 |  |  | N_Shore | 3.3 |
| cg06628792 | 0.32129 | 83 | 0.0981 | 96 | Y | TTTY14 | NR_001543 | Island | 3.3 |
| cg22999025 | 0.47988 | 92 | 0.14848 | 17 | 17 |  |  |  | 3.2 |
| cg06817454 | 0.35249 | 7381 | 0.10938 | 6999 | 1 |  |  |  | 3.2 |
| cg27636129 | 0.43325 | 76 | 0.13496 | 16 | Y | SRY | NM_003140 | N_Shore | 3.2 |
| cg01543933 | 0.47542 | 314 | 0.14848 | 17 | 17 | CYTSB | NM_152904 |  | 3.2 |
| cg10718795 | 0.43042 | 76 | 0.13481 | 111 | X |  |  | S_Shore | 3.2 |
| cg10166664 | 0.45769 | 4817 | 0.14901 | 4162 | 5 |  |  | N_Shelf | 3.1 |
| cg16719099 | 0.55744 | 136 | 0.18284 | 161 | X | MIR505 | NR_030230 |  | 3.0 |
| cg08152839 | 0.33463 | 877 | 0.11082 | 944 | 15 | ST8SIA2 | NM_006011 | N_Shore | 3.0 |
| cg10202113 | 0.8635 | 16382 | 0.29435 | 14689 | 1 |  |  | S_Shore | 2.9 |
| cg03959986 | 0.30413 | 530 | 0.10686 | 235 | 17 | SMG6 | NM_001170957 | Island | 2.8 |
| cg01900066 | 0.42244 | 87 | 0.1486 | 295 | Y | EIF1AY | NM_004681 |  | 2.8 |
| cg01037683 | 0.43042 | 76 | 0.15375 | 37 | 17 | CYTSB | NM_152904 | N_Shelf | 2.8 |
| cg13568515 | 0.40924 | 342 | 0.14626 | 249 | 9 | JAK2 | NM_004972 | Island | 2.8 |
| cg01311227 | 0.32941 | 359 | 0.11877 | 201 | Y | RPS4Y1 | NM_001008 |  | 2.8 |
| cg13413719 | 0.39092 | 9080 | 0.14204 | 7996 | 6 |  |  |  | 2.8 |
| cg15544633 | 0.38198 | 208 | 0.14075 | 396 | 2 | LIPT1 | NM_145198 | Island | 2.7 |
| cg11684211 | 0.4158 | 71 | 0.15418 | 79 | Y | TTTY14 | NR_001543 | Island | 2.7 |
| cg14273923 | 0.47508 | 91 | 0.17763 | 267 | Y |  |  | Island | 2.7 |
| cg02980566 | 0.38263 | 171 | 0.14593 | 126 | 17 |  |  | N_Shelf | 2.6 |
| cg01426558 | 0.44747 | 106 | 0.17165 | 162 | Y | DDX3Y | NM_001122665 | N_Shore | 2.6 |
| cg22760086 | 0.3217 | 258 | 0.12384 | 152 | 17 | TMEM11 | NR_024547 | Island | 2.6 |
| cg21897425 | 0.30856 | 105 | 0.12028 | 159 | 17 |  |  | Island | 2.6 |
| cg03908845 | 0.37067 | 689 | 0.14479 | 604 | X | MCF2 | NM_005369 |  | 2.6 |
| cg08357313 | 0.52143 | 109 | 0.20505 | 110 | Y | RBMY1A3P | NR_001547 |  | 2.5 |
| cg00504457 | 0.32876 | 930 | 0.12985 | 916 | 19 | MCOLN1 | NM_020533 | Island | 2.5 |
| cg00063477 | 0.5653 | 130 | 0.23071 | 155 | Y | EIF1AY | NM_004681 | S_Shelf | 2.5 |
| cg08433110 | 0.64745 | 184 | 0.27582 | 90 | 6 | GMDS | NM_001500 |  | 2.3 |
| cg20865618 | 0.39782 | 192 | 0.17219 | 151 | 7 |  |  | S_Shore | 2.3 |
| cg05544622 | 0.30745 | 187 | 0.13475 | 214 | Y | TSPY1 | NM_003308 | Island | 2.3 |
| cg25578822 | 0.32754 | 294 | 0.14371 | 213 | 6 | BRD2 | NM_001113182 | N_Shore | 2.3 |
| cg01389203 | 0.33108 | 1305 | 0.14722 | 1109 | 6 |  |  | S_Shore | 2.2 |
| cg10065520 | 0.39766 | 143 | 0.17718 | 149 | 17 | MAP2K3 | NM_002756 | N_Shelf | 2.2 |
| cg14456004 | 0.41266 | 791 | 0.18445 | 946 | 13 |  |  | Island | 2.2 |
| cg02522936 | 0.43884 | 78 | 0.19859 | 25 | Y | TTTY10 | NR_001542 |  | 2.2 |
| cg03610148 | 0.63383 | 173 | 0.28893 | 151 | 3 | FHIT | NM_001166243 |  | 2.2 |
| cg00578645 | 0.36882 | 272 | 0.16833 | 276 | X |  |  | N_Shore | 2.2 |
| cg17598552 | 0.34626 | 12149 | 0.16238 | 11978 | 6 | PSORS1C1 | NM_014068 |  | 2.1 |
| cg01762011 | 0.48826 | 267 | 0.23016 | 235 | X |  |  | Island | 2.1 |
| cg04246167 | 0.36987 | 14853 | 0.17647 | 11774 | 3 | C3orf67 | NM_198463 |  | 2.1 |
| cg08476890 | 0.65639 | 316 | 0.3141 | 122 | X | MCF2 | NM_001099855 | Island | 2.1 |
| cg02884332 | 0.31231 | 440 | 0.14975 | 439 | Y | EIF1AY | NM_004681 | Island | 2.1 |
| cg00007215 | 0.35393 | 213 | 0.17003 | 191 | 17 |  |  | Island | 2.1 |
| cg26928789 | 0.42282 | 280 | 0.20444 | 26 | Y | TTTY10 | NR_001542 |  | 2.1 |
| cg05370853 | 0.4671 | 146 | 0.22702 | 29 | 6 | HLA-DQA1 | NM_002122 |  | 2.1 |
| cg27325772 | 0.35671 | 117 | 0.1743 | 21 | Y |  |  | S_Shelf | 2.0 |
| cg19244032 | 0.44432 | 80 | 0.21945 | 339 | Y | NLGN4Y | NR_028318 | Island | 2.0 |
| cg04209650 | 0.31538 | 109 | 0.1569 | 40 | 17 | ALDH3A1 | NM_001135167 | Island | 2.0 |
